# PROFILE OF DIRECT CARE WORKERS ACROSS THE LONG-TERM CARE SERVICES AND SUPPORTS SECTOR

**DOI:** 10.1093/geroni/igae098.1987

**Published:** 2024-12-31

**Authors:** Christopher Kelly, Candace Kemp, Jennifer Craft Morgan

**Affiliations:** University of Nebraska Omaha, Omaha, Nebraska, United States; Georgia State University, Atlanta, Georgia, United States; Georgia State University, Atlanta, Georgia, United States

## Abstract

DCWs across long-term care are predominately women, people of color and disproportionally immigrants. About half have health insurance through their employer and about a quarter get health insurance through Medicaid or another means tested program. About half live under 200% of the poverty line. DCWs also face dangerous working conditions, persistent occupational segregation, have limited access to paid leave, and experience very little career advancement. As stated by Scales & Lepore (2020) “[direct care work] requires a mix of technical caregiving skills; health-related knowledge; infection prevention and control expertise; emotional intelligence and relational skills; and problem-solving and decision-making abilities, among other competencies (p. 173).” Despite highly meaningful jobs with high intrinsic rewards, the lack of extrinsic rewards including compensation, drive turnover. Turnover rates in long-term care have been persistently slow to recover since the start of the pandemic and the recovery has been most difficult for women and people of color. In this context, the use of agency staff, or those that are temporarily hired from staffing agencies to fill staffing shortages, has remained persistently high. Use of agency staff makes relationship-based, person-centered care difficult. Many residential care organizations have reduced the number of new residents because they do not have the staffing to accommodate them despite having available licensed beds. Using data from the 2020-2022 American Community Survey we examine trends among direct care workers (DCWs) in terms of job quality, occupation and industry. Persistent issues with churn, lack of career advancement, low wages, and few benefits remain.

